# Mechanisms Underlying the Environmentally Induced Plasticity of Leaf Morphology

**DOI:** 10.3389/fgene.2018.00478

**Published:** 2018-10-24

**Authors:** Michael André Fritz, Stefanie Rosa, Adrien Sicard

**Affiliations:** ^1^Institut für Biochemie und Biologie, Universität Potsdam, Potsdam, Germany; ^2^Department of Plant Biology, Swedish University of Agricultural Sciences and Linnean Center for Plant Biology, Uppsala, Sweden

**Keywords:** plants, leaf morphology, environment, developmental plasticity, gene regulatory networks, sensory system, gene responsiveness

## Abstract

The primary function of leaves is to provide an interface between plants and their environment for gas exchange, light exposure and thermoregulation. Leaves have, therefore a central contribution to plant fitness by allowing an efficient absorption of sunlight energy through photosynthesis to ensure an optimal growth. Their final geometry will result from a balance between the need to maximize energy uptake while minimizing the damage caused by environmental stresses. This intimate relationship between leaf and its surroundings has led to an enormous diversification in leaf forms. Leaf shape varies between species, populations, individuals or even within identical genotypes when those are subjected to different environmental conditions. For instance, the extent of leaf margin dissection has, for long, been found to inversely correlate with the mean annual temperature, such that Paleobotanists have used models based on leaf shape to predict the paleoclimate from fossil flora. Leaf growth is not only dependent on temperature but is also regulated by many other environmental factors such as light quality and intensity or ambient humidity. This raises the question of how the different signals can be integrated at the molecular level and converted into clear developmental decisions. Several recent studies have started to shed the light on the molecular mechanisms that connect the environmental sensing with organ-growth and patterning. In this review, we discuss the current knowledge on the influence of different environmental signals on leaf size and shape, their integration as well as their importance for plant adaptation.

## Introduction

The variability of forms of life on Earth has long captivated the attention of biologists. However, no less striking is the variability potentially available within the same species. It is now well established that the same genotype is often capable of giving rise to different phenotypes when exposed to different environmental conditions. This phenotypic plasticity, which is often expressed as a reaction norm representing the relationship between phenotypes and environmental variables, is known to be genetically determined and is, therefore, likely to be subjected to selective pressures (Bradshaw, 1965; West-Eberhard, 2003; Gratani, 2014; Sultan, 2017). Flexible phenotypes allow to conserve an adaptive potential that may be crucial for surviving in heterogeneous environments but they, also, offer the possibility of ‘fixing’ different phenotypic means adapted to specific habitats during colonization (Schlichting, 1986; Stearns, 1989; Moczek et al., 2011). Plasticity, itself, may also evolve as a change ‘in the shape of reaction norm’ or as the emergence of a new environmentally induced phenotype (Scheiner, 1993; Serobyan and Sommer, 2017; Sultan, 2017). Natural selection may have, therefore, favored the evolution of sensory systems allowing organisms to sense their environment and modify their development and physiology accordingly. These signals have, nevertheless, to be integrated into Gene Regulatory Networks (GRNs) controlling functional traits in such a way that they induce rapid and adapted changes in phenotypes. Physiological plasticity was proposed to facilitate adaptation to rapidly fluctuating environments while morphological variations may have as a longer-term function most likely to respond to average seasonal conditions (Gratani, 2014).

**FIGURE 1:**
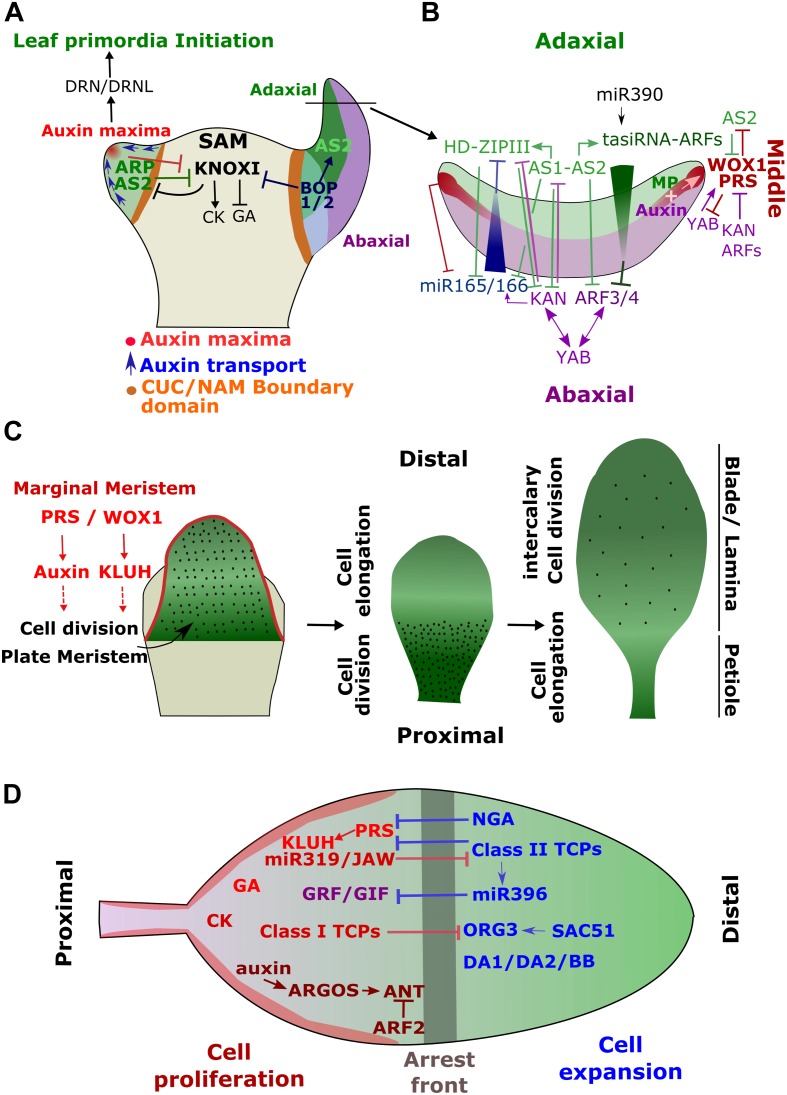
Simplified model of the Genetic Regulatory Networks (GRN) controlling leaf development. **(A)** Leaves initiate from the flank of the shoot apical meristem (SAM) upon the formation of an auxin maxima caused by the cellular repolarisation of the auxin efflux carrier PIN1. This together with the activation of *ARP* will repress genes involved in the maintenance of SAM (*KNOXI* genes) at the site of leaf primordia initiation. The primordia will outgrow by increasing cell divisions through the activity of DRN and DRNL. **(B)** Early after its initiation, the primordia will acquire its adaxial-abaxial polarity through the activation of a complex GRN involving multiple negative feedback mechanisms. miR166 represses the adaxial HD-ZIPIII genes in the abaxial domain and miR390 induces tasiRNAs restricting ARF3 and 4 to the abaxial side. miR166 is itself inhibited by AS1-AS2 in the adaxial end where AS1-AS2 promote the tasiRNA-ARFs. *AS1-AS2* are, in turn, inhibited by KAN in the abaxial domain. The establishment of the adaxial-abaxial polarity contributes to activate *WOXs* genes (*WOX1* and *PRS*) in the medial domain, which result in the formation of the mediolateral axis. **(C)** Schematic representation of the main developmental processes involved in leaf growth. PRS and WOX1 activate *KLUH* at the margin of the leaf primordia (or marginal meristem). KLUH, in turn, promotes, in a non-cell autonomous manner, cell proliferation in the center of the leaves (or plate meristem). Later, cell divisions will be restricted to the proximal part of the leaves for several days while cell elongation will be initiated at the distal end. Subsequently, cell divisions will only continue at intercalary meristems before they completely stop. At this stage, leaves will mainly grow through cell elongation. **(D)** The cell division arrest front is established through the activation of the *Class II TCPs* at the distal end of the leaf primordia, where they will together with *NGA* inhibit *PRS* expression. Class II TCPs also activate miR396, which represses *GRF/GIF* function in the distal end. In the proximal side, miR319/JAW prevents *Class II TCPs* function.

When environmental changes occur, plants do not have the possibility to move to more favorable conditions. Phenotypic plasticity is therefore likely to be prevalent in plants and to be fundamental to maintain an optimal fitness when environmental conditions fluctuate or upon exposure to transitory harmful conditions (Schlichting, 1986; Gratani, 2014). Leaves are a crucial interface between plants and the environment (Tsukaya, 2005; Nicotra et al., 2011). They allow plants to capture sunlight, exchange gasses with the atmosphere and to regulate their temperature. Leaves are the main photosynthetic organ allowing plants to accumulate organic nutrients for optimal growth and abundant seed set (Gifford et al., 1984; Mohr and Schopfer, 1995; Terashima et al., 2011; Koester et al., 2014; Weraduwage et al., 2015). Typical leaves in flowering plants have a flat laminar structure constituted by a small stem, the petiole and a broad blade, which is also called the lamina (Figure 1) (Kalve et al., 2014b). The petiole is mostly constituted of vascular tissues that transport water and nutrients. The blade structure is more complex and made of three main tissues; the epidermis, mesophyll and vascular tissue (Kalve et al., 2014b). The epidermis serves as a protective layer controlling for instance water evaporation (Becraft, 1999). The epidermis is perforated by specialized structures, the stomata, constituted of microscopic pores surrounded by two guard cells, which together regulate the diffusion of gasses with the atmosphere as well as water loss by transpiration (Terashima et al., 2011; Lau and Bergmann, 2012). The mesophyll is especially rich in chloroplasts and constitutes the primary photosynthetic tissue in plants.

The shape and size of the leaf blade and petiole, as well as the density of stomata, have been shown to be extremely variable in plants (Bar and Ori, 2014; Chitwood and Sinha, 2016; Tsukaya, 2018). These parameters vary among species, populations and individuals but also within the same genotype. In the latter case, it can vary within the lifetime of the plant, a process known as heteroblasty, or between environments (Tsukaya, 2005; Zotz et al., 2011). Some plants species have, even, evolved the ability to develop completely different leaf types depending on their growing conditions, a phenomenon known as heterophylly (Nakayama et al., 2017). The timing of heteroblastic changes, i.e., heterochrony, can be modified during evolution or as a response to environmental changes (Chitwood et al., 2012; Cartolano et al., 2015). It is easy to understand why leaf morphology may be very plastic with regards to environmental conditions (Nicotra and Davidson, 2010). Plants may, for instance, prefer to develop broad lamina to maximize light capture (Weraduwage et al., 2015). But, on the other hand, if the sunlight is too intense, a large exposure to the solar radiation may lead to overheating (Fetcher, 1981; Ort, 2001). The thin and large structure of leaves is also highly sensitive to mechanical stress such as strong wind (Gardiner et al., 2016). The overall shape and size of the leaves need therefore to be controlled depending on the surrounding conditions in order to optimize the surface for gas exchange and the amount of light that can be captured by photosynthesis while minimizing environmental stresses.

Plastic phenotypic responses rely on the ability of organisms to modulate GRNs and often in a reversible manner (Beaman et al., 2016). Several molecular mechanisms may be prone to contribute to such dynamic responses. Phytohormones have key functions in almost all aspect of plant development and can act as long-range molecular signals (Alabadí et al., 2009; Wolters and Jürgens, 2009; Vanstraelen and Benková, 2012; Khan et al., 2013; Alazem and Lin, 2015; Schaller et al., 2015; Druege et al., 2016; Eremina et al., 2016; Lacombe and Achard, 2016; Campos-Rivero et al., 2017; Qi and Torii, 2018; Tian et al., 2018). Environmentally induced changes in hormone concentration and/or sensitivity can, therefore, promote coordinated developmental responses (Wolters and Jürgens, 2009; Eremina et al., 2016; Campos-Rivero et al., 2017; Yang and Li, 2017; Qi and Torii, 2018). Chromatin structure, including DNA methylation, the covalent modification of histone N-terminal tails (i.e., histone marks) and the incorporation of histone variants, has also been shown to play a critical role in modulating gene transcription in response to environmental variables (Berr and Shen, 2010; Baulcombe and Dean, 2014; Pikaard et al., 2014; Lämke and Bäurle, 2017; Hewezi, 2018). Dynamic changes in chromatin state are believed to be particularly important to promote phenotypic plasticity mostly because they allow both transitory and rapid changes in gene expression without changes in the DNA sequence (Duncan et al., 2014; Schlichting and Wund, 2014). Depending on the type of modifications, newly established chromatin states can be perpetuated during DNA replication and even passed on to the next generation. In plants, changes in chromatin state regulate several developmental transitions, organogenesis processes, cell fate establishment as well as responses to environmental cues (Herman and Sultan, 2011; Baulcombe and Dean, 2014; Morao et al., 2016; Fortes and Gallusci, 2017; Köhler and Springer, 2017; Friedrich et al., 2018). Small RNAs (sRNA) are also regulating many aspects of plant development and physiology (Li and Zhang, 2016; D’Ario et al., 2017). Several classes of sRNA generated through different RNA processing pathways have been identified. They regulate the activity of key regulators by mediating the cleavage of complementary mRNA or by inducing chromatin modifications at their target loci (Borges and Martienssen, 2015). Because sRNAs act in a sequence specific manner and are able to diffuse across cells and organs, they act as inhibitory signals able to modulate the activity of GRNs (Borges and Martienssen, 2015; Kehr and Kragler, 2018; Reagan et al., 2018). Because of these properties, sRNAs are often involved in the establishment of spatial and temporal borders between developmental processes, and their function can be regulated at the level of their expression, biogenesis and interaction as well as through the control of the expression of their target genes (Morao et al., 2016). Several of these regulatory mechanisms are influenced by environmental variables and contribute to adjust phenotype to the surrounding conditions (Zhang, 2015; Wang and Chekanova, 2016).

Here, after a brief synopsis on leaf development, we review the current knowledge on the molecular mechanisms underlying the plasticity of leaf morphology and how this may contribute to improving plant fitness in fluctuating environments. We mainly focus on the leaves of flowering plants with the aim to highlight the molecular features behind environmental sensing and the integration of the subsequent signals into a comprehensive developmental decision. Taking into account the scope of this review, we have limited our discussion to the influence of abiotic signals on leaf morphology for which the associated molecular mechanisms have been described in more detail.

## Synopsis of the Genetic Control of Leaf Morphogenesis

In this part, we described the main regulatory nodes controlling different aspects of leaf development with the aim to introduce the gene regulatory modules integrating environmental signals and their effect on leaf development. The complexity of the genetic network controlling leaf growth has been discussed elsewhere (for more detailed information please refer to Gonzalez et al., 2012; Lau and Bergmann, 2012; Pillitteri and Torii, 2012; Hepworth and Lenhard, 2014; Kalve et al., 2014b; Rodriguez et al., 2014; Czesnick and Lenhard, 2015; Ichihashi and Tsukaya, 2015; Du et al., 2018; Eng and Sampathkumar, 2018; Maugarny-Calès and Laufs, 2018; Tsukaya, 2018; Zoulias et al., 2018).

### Leaf Primordia Initiation and Outgrowth

Leaves are initiated as primordia at the flank of the Shoot Apical Meristem (SAM) – an indeterminate structure containing a pool of pluripotent cells at the origin of all aerial plant organs (Figure 1A) (Braybrook and Kuhlemeier, 2010; Tameshige et al., 2016; Du et al., 2018; Maugarny-Calès and Laufs, 2018). The recruitment of leaf founder cells within the peripheral zone of the SAM is mediated by the formation of a concentration maxima of the plant hormone auxin (Reinhardt et al., 2003; Heisler et al., 2005). The auxin efflux carrier PIN-FORMED1 (PIN1) dynamically repolarizes at the cellular level during SAM growth creating convergent flows of auxin at specific positions. The gradient of auxin whithin the SAM is furhter reinforced and stabilized by the local activity of the auxin influx transporters, AUXIN RESISTANT 1 (AUX1) and Like-AUXs (LAX 1, 2, and 3) (Bainbridge et al., 2008). The resulting raise in auxin concentration will locally repress the expression of genes involved in the maintenance of the SAM including the class-1 Knotted-like homeobox (*KNOXI*) genes, *SHOOTMERISTEMLESS* (*STM*) and *BREVIPEDICELLUS* (*BP*) (Heisler et al., 2005; Hay, 2006). This repression is further reinforced by the activation of the MYB domain transcription factor ASYMMETRIC LEAVES1 (AS1), (also known as ROUGHSHEATH2 in maize or PHANTASTICA in *Antirrhinum majus*, and collectively termed ARP) (Timmermans, 1999; Tsiantis, 1999; Byrne et al., 2000; Eckardt, 2004). AS1 interacts with the LATERAL ORGAN BOUNDARIES DOMAIN protein, ASYMMETRIC LEAVES2 (AS2*)* and bind directly to the promoter of *KNOXI* genes leading to their stable epigenetic silencing through the recruitment of the Polycomb Repressive Complex 2 (PRC2) (Luo et al., 2012; Lodha et al., 2013). The formation of auxin gradient within the SAM also contributes to the formation of boundary domains constituted by slow dividing cells which separate the outgrowing primordia from the rest of the meristem (Breuil-Broyer et al., 2004; Reddy, 2004; Łabuz et al., 2015). These domains are maintained by the activity of several factors including members of the transcription factor family NO APICAL MERISTEM/CUPSHAPED COTYLEDON (NAM/CUC) (Aida, 1997; Hibara et al., 2006). KNOXI transcription factors maintain the meristematic activity in SAM through the regulation of hormonal pathways. They promote cytokinin (CK) production, which maintains cell proliferation while preventing cell differentiation through the inhibition of gibberellic acid (GA) signaling (Hay et al., 2002; Jasinski et al., 2005). The repression of *KNOXI* induces, therefore, a local change in hormonal status at the site of primordia initiation, which may contribute to accelerate and re-orientate cell divisions promoting leaf primordia outgrowth. In addition, auxin maxima activate the expression of the Ethylene Response Factors *DORNRÖNSCHEN* (*DRN*) and *DRN*-*LIKE* (*DRNL*) [their single ortholog in tomato is known as *LEAFLESS* (*LFS*)] which play an essential role in promoting cell proliferation at the site of primordia emergence (Chandler et al., 2011; Seeliger et al., 2016; Capua and Eshed, 2017). Primordia outgrowth is also facilitated by the remodeling of the cell wall mechanical properties that become more extensible (Peaucelle et al., 2011).

### Leaf Polarity Establishment

Shortly after its emergence, the leaf primordia will rapidly become asymmetric and acquire different polarity axes (Figure 1B). In fact, the adaxial-abaxial polarity axis is established very early in response to a mobile signal emanating from the SAM, known as the Sussex signal, prior to the emergence of leaf primordia (Sussex, 1954; Caggiano et al., 2017; Yu et al., 2017). The adaxial cell fate is promoted by the expression of the HD-ZIPIII transcription factors, *REVOLUTA* (*REV*), *PHAVOLUTA* (*PHV*) and *PHABULOSA* (*PHB*) (McConnell et al., 2001; Emery et al., 2003). Their expression is restricted to the adaxial side of the primordia by a gradient of the microRNAs miR165/166, established from the abaxial end (Yao et al., 2009; Skopelitis et al., 2012, 2017; Tatematsu et al., 2015). The expression of the precursor of these microRNAs is in turn inhibited in the adaxial side by the HD-ZIPIII transcription factors and their downstream targets (Bou-Torrent et al., 2012; Merelo et al., 2016). During primordia outgrowth, AS2 expression will also become restricted to the adaxial side and contributes to specifying its fate (Iwakawa et al., 2002; Lin et al., 2003). Similarly, the abaxial boundary is also maintained by a gradient of small RNAs and their targets. The abaxial fate is promoted by members of the transcription factors families KANADI (including KAN1 and KAN2) and AUXIN RESPONSE FACTORS (ARF 2, 3 and 4), which promote the transcription of genes involved in the maintenance of the abaxial identity the YABBY transcription factors; FIL, YAB2 and YAB3 (Sawa et al., 1999; Siegfried et al., 1999; Pekker, 2005; Guan et al., 2017; Yu et al., 2017). YABs sustained the adaxial-abaxial polarity most likely by directly promoting the expression of *KAN1* and *ARF4* (Bonaccorso et al., 2012). KANs and HD-ZIPIII antagonize each other and inversely regulate the expression of genes involved in auxin transport and biosynthesis (Eshed, 2004; Huang et al., 2014). The expression of the ARF genes is restricted to the abaxial side by miR390, which induces trans-acting short interfering RNAs targeting the ARFs (tasiRNA-ARFs) in the adaxial domain (Fahlgren et al., 2006; Hunter, 2006; Iwasaki et al., 2013). AS1-AS2 protein complex contributes to the boundary formation by negatively regulating ARFs and miR166A in the adaxial domain while directly promoting the expression of the tasiRNA-ARF precursor, *TSA3A* (Iwasaki et al., 2013; Husbands et al., 2015). KAN1 in contrary activates miR166 in the abaxial domain (Merelo et al., 2016). These complex genetic interactions together with a threshold-based readout mechanism of small RNAs gradients allows the formation of a sharp and robust boundary within the adaxial-abaxial axis (Skopelitis et al., 2017).

The establishment of this polarity will also contribute to defining the mediolateral axis. Indeed, as mentioned above, the adaxial and abaxial genes will regulate auxin biosynthesis resulting in higher auxin level in the abaxial domain (Qi et al., 2014; Guan et al., 2017). The overlap at the adaxial-abaxial boundary (i.e., middle domain) between a high abaxial auxin concentration and the adaxial expression of the *ARF*, *MONOPTEROS* (*MP*) results in a higher auxin response and the activation of the *WUSCHEL-RELATED HOMEOBOX* (*WOX*) genes, *WOX1* and *PRESSED-FLOWER* (*PRS*) (Guan et al., 2017; Qi et al., 2014). The expression of WOX1 and PRS is, therefore, restricted to the middle domain but with a stronger expression in the marginal region, often defined as the ‘marginal meristem’ (Figures 1B,C) (Alvarez et al., 2009, 2016; Nakata et al., 2012; Guan et al., 2017; Tsukaya, 2018). WOX1 and PRS will activate the expression of *KLUH*, which encodes a cytochrome P450 CYP78A5 monooxygenase promoting cell division through a non-cell autonomous mechanism in the central part of the developing leaf, known as the ‘plate meristem’ (Anastasiou et al., 2007; Nakata et al., 2012; Tsukaya, 2018). Auxin also plays an important role in promoting lamina outgrowth and *WOX* genes have been shown to promote auxin biosynthesis (Tadege et al., 2011; Wang et al., 2011). These regulatory pathways together with the specification of the different leaf domain will promote the outgrowth of leaf blade along the mediolateral axes. The alteration of hormonal homeostasis together with the function of the polarity factors will promote dynamic alterations in cell wall mechanical properties early during leaf development by inducing local changes in the methyl-esterification status of the cell-wall pectins (Qi et al., 2017). This results in a higher cell wall elasticity in the middle domain along the adaxial/abaxial axis, which in turn promotes the asymetric growth and the flattening of the leaf primordia and also contributes to maintaining polarity through a feedback mechanism regulating the expression of polarity factors (Qi et al., 2017). The medio-lateral polarity is reinforced by several regulatory mechanisms. The *YABBY* genes contribute to activate the expression of *WOX1* in the middle domain (Nakata et al., 2012). *WOX* expression is, in turn, spatially regulated by the abaxial factors KAN and ARF3/4 and the adaxial gene, *AS2* (Alvarez et al., 2016; Guan et al., 2017). PRS and WOX1 contribute to maintain the adaxial/abaxial patterning by restricting the expression of AS2 and FIL factors to the adaxial and abaxial domains, respectively. They also regulate HD-ZIPIII expression through the inhibition of *miR165/166* in the marginal domain (Nakata et al., 2012).

Lastly, a proximal-distal axis is also defined. The BTB/POZ domain and ankyrin repeat genes *BLADE ON PETIOLE1* (*BOP1*) and *BOP2* are expressed in the proximal region of the leaf primordia where they repress leaf blade outgrowth and specify the petiole (Ha et al., 2004). BOP1 and 2 also contribute to activate adaxial genes such as *AS2* and to repress *KNOXI* in the leaf primordia (Ha et al., 2007).

### Leaf Size Control

After primordia initiation, leaves grow according to two main processes (Figure 1C). Cells within the leaf primordia first undergo a series of cell divisions before switching into a phase of postmitotic cell expansion. Therefore, the final leaf size will be determined by both the number and size of cells. Any processes affecting the rate, duration and/or spatial distribution of these phases will influence the final size of the leaves (Tsukaya, 2018). The genetic and hormonal pathways controlling these phases have been studied in great detail and many important regulators have been identified (reviewed in Tsukaya, 2006; Hepworth and Lenhard, 2014; Kalve et al., 2014a; Czesnick and Lenhard, 2015).

In the plant model *Arabidopsis thaliana*, the switch between cell division and cell elongation follows a proximal-distal gradient. Cells at the proximal end divide until they reach a certain position along the basipetal axes, from which they will stop dividing and start expanding (Figure 1C) (Czesnick and Lenhard, 2015). This ‘arrest front’ is established early on, remains at a constant distance from the proximal end until cell divisions completely stop (Kazama et al., 2010; Andriankaja et al., 2012). Nevertheless, few epidermal cells, the meristemoid cells, continue to divide for some time contributing substantially to the final leaf size (Geisler, 2000). While the patterning of these growth processes varies among flowering plant species, their main gene regulatory networks appear to be conserved (Figure 1D) (Das Gupta and Nath, 2015; Tsukaya, 2018).

Consistently with the emergence of a ‘cell division arrest front,’ the marginal expression of *PRS* will become restricted to the proximal part of the leaf (Alvarez et al., 2016). In the distal end, its expression is inhibited by the redundant function of the class-II TEOSINTE BRANCHED1/CYCLOIDEA/PROLIFERATING CELL FACTORs (TCPs) and the NGATHA (NGA) transcription factors (Alvarez et al., 2016). This repression contributes to promote cell elongation and inhibit cell division. The function of the TCPs is prevented in the proximal region by miR319/JAW, a microRNA targeting their transcripts (Palatnik et al., 2003; Ori et al., 2007). On the other hand, cell divisions are maintained in the plate meristem through the activity of the GROWTH REGULATOR FACTORs (GRFs) and their interacting partners GRF-INTERACTING FACTOR1/ANGUSTIFOLIA3 (GIF1/AN3), GIF2, and GIF3 and the regulation of *CYCLIN B* (*CYCB*) expression (Horiguchi et al., 2005; Lee et al., 2009; Rodriguez et al., 2010; Debernardi et al., 2014). TCP4 activates miR396 at the distal end which targets and cleave most of the *GRF* mRNAs restricting GRFs function to the proximal region (Rodriguez et al., 2010). *AN3* is restricted to the plate meristem where it strongly influences on the maintenance of cell proliferation by recruiting the chromatin remodeling complexes SWITCH/SUCROSE NON-FERMENTING (SWIF/SNF) to its target to regulate their expression (Vercruyssen et al., 2014). AN3 protein accumulates in a gradient along the proximal-distal axes and its intracellular concentration strongly correlates with cell proliferation activity (Kawade et al., 2017). While many GRFs positively regulate cell division, others have been shown to limit cell proliferation. For instance, *GRF9* restricts cell proliferation during early leaf primordia outgrowth by directly interacting with the promoter of the basic helix-loop-helix (bHLH) transcription factor *OBP3-RESPONSIVE GENE 3* (*ORG3*) (Omidbakhshfard et al., 2018). *ORG3* was also shown to be directly and antagonistically regulated by the class-I TCP, *TCP20* and the bHLH gene *SAC51*, which is known to promote cell elongation (Imai et al., 2006; Andriankaja et al., 2014). It was therefore proposed that TCP20 maintains low levels of ORG3 to stimulate cell proliferation while SAC51 would counteract this repression by activating *ORG3* and promoting the transition toward cell elongation (Andriankaja et al., 2014). The *KLUH* derived non-cell autonomous signal also plays a primordial role in maintaining cell proliferation during leaf growth (Anastasiou et al., 2007; Eriksson et al., 2010; Nakata et al., 2012). *KLUH* derived signal has been recently shown to regulate the incorporation of the histone variant H2A.Z at a key locus during germ-line specification suggesting that its effect on organ size could also be mediated by a chromatin based mechanism (Zhao et al., 2018). Other factors have also shown to regulate the timing of the cell proliferation period (Gonzalez et al., 2012; Hepworth and Lenhard, 2014; Czesnick and Lenhard, 2015). For instance, factors regulating the protein stability of key regulators of cell division also contribute to modulate the duration of the cell proliferation period. The ubiquitin binding protein DA1 acts synergistically with the E3 ubiquitin ligases, DA2 and BIG BROTHER (BB), to target factors promoting cell division for degradation by the proteasome (Disch et al., 2006; Li et al., 2008; Xia et al., 2013; Du et al., 2014).

Hormonal signaling appears to also play an important role in regulating leaf size and in maintaining cell division (Wolters and Jürgens, 2009; Czesnick and Lenhard, 2015; Maugarny-Calès and Laufs, 2018). GA stimulates cell division through the repression of cell cycle inhibitors such as KIP-RELATED PROTEIN 2 (KRP2) and SIAMESE (SIM) (Achard et al., 2009). Auxin promotes the expression of the AP2 transcription factor gene, *AINTEGUMENTA* (*ANT*) through the activation of *ARGOS* leading to an up-regulation of a cell cycle activator, the D type cyclin *CYCD3;1* (Krizek, 1999; Mizukami and Fischer, 2000; Hu, 2003). Brassinosteroids (BR) have been shown to stimulate cell division through the up-regulation of cell cycle activators (Zhiponova et al., 2013). And finally, cytokinins have also been proposed to induce cell proliferation (Shani et al., 2010). However, the integration of hormonal signals is complex and most likely context dependent, since many of these hormones have also been shown to also promote cell elongation. For instance, the auxin response gene, *ARF2* whose activity is also positively regulated by BR, has been shown to down-regulate *ANT* and *CYCD3;1* promoting the transition toward the cell expansion phase (Schruff, 2005; Vert et al., 2008).

The differentiation of the photosynthetic apparatus at the distal part of the leaf is believed to initiate the signal that will trigger the arrest of cell proliferation and the onset of cell elongation (White, 2006). Meristemoid cells will continue to divide for longer until the progression of a secondary division arrest front. This is mediated by two DNA-binding proteins, PEAPOD1 (PPD1) and PPD2 which act in a transcriptional repressor complex suppressing the expression of genes promoting meristemoid division (White, 2006; Li et al., 2018). The activity of this complex is counterbalanced by the F-box protein STERILE APETALA (SAP) which promotes the degradation of PPD1 and 2 as well as KIX8/9 which serve as an adaptor protein between PPDs and the transcriptional repressor TOPLESS (TPL) (Wang et al., 2016; Li et al., 2018).

Once cell divisions stops, cells will start to elongate by increasing turgor pressure through water uptake in the vacuole and induce cell wall remodeling to sustain the increase in volume (Schopfer, 2006; Kalve et al., 2014a). These modifications include an auxin- and brassinolide-induced acidification of the apoplast through the activation of the H+-ATPases leading to the induction of EXPANSINs (EXPs) (Hager, 2003; Cosgrove, 2005). EXPs in turn promote cell elongation by inducing the loosening of the cell wall through the destruction of the hydrogen bonds between polysaccharides (Cho and Cosgrove, 2000; Goh et al., 2014). Mechanisms controlling the integrity of the cell wall also feedback on the cellular growth, a process involving a family of membrane-spanning receptor-like kinase (RLKs) including THESEUS1 and its close homolog FERONIA (Hématy et al., 2007; Guo et al., 2009). This process is accompanied by successive endoreduplication cycles, an altered cell cycle in which the DNA is duplicated without any mitosis leading to an increase in the DNA content (Kalve et al., 2014a; Orr-Weaver, 2015; Scholes and Paige, 2015). The increase in ploidy often correlates with an increase in cell size and significantly contribute to the final organ size (Sugimoto-Shirasu and Roberts, 2003; del Pozo et al., 2006). Many regulatory pathways have been shown to regulate these processes highlighting notably a predominant role of different hormonal signaling (reviewed in Wolters and Jürgens, 2009; Vanstraelen and Benková, 2012; Kalve et al., 2014a). As discussed below, cell elongation is also highly connected to environmental conditions and to the nutritional status of the plant. For instance, the kinases TARGET Of RAPAMYCIN (TOR) stimulates growth under nutrient-limiting conditions by modulating the translational activity of ribosomes (Deprost et al., 2007; Ren et al., 2011). TOR signaling has also been shown to be activated by auxin (Schepetilnikov et al., 2013).

### Stomata Patterning

During leaf development, stomata will differentiate from the meristemoids in a basipetal manner (Zoulias et al., 2018). Few protodermal cells will transition into meristemoid mother cells, which will then undergo a self-renewing asymmetric division leading to the formation of a meristemoid and a daughter cell. The meristemoid can undergo additional rounds of division or differentiate into a guard mother cell (GMC). The latter will then complete its differentiation into stomata through an additional symmetrical cell division event. The transitions toward the different steps of stomata differentiation are controlled by a series of basic-helix-loop-helix (bHLH) transcription factors including SPEECHLESS (SPCH), MUTE, and FAMA (Ohashi-Ito and Bergmann, 2006; MacAlister et al., 2007; Pillitteri et al., 2007). They are expressed transiently and activate developmental programs that will determine the fate of protodermal cells as well as control the number of amplifying divisions and, thus, the spacing between each stomata. The function of these genes is dependent on a second class of bHLH proteins, the INDUCER OF CBF EXPRESSION1/SCREAM (ICE1/SCRM) and SCRM2 (Kanaoka et al., 2008). The inhibition of cell proliferation after the symmetrical division of the GMC depends on two MYB transcription factors, FOUR LIPS (FLP) and MYB88 as well as on the inhibition of CYCA2.3 and CDKB1;1 (Lai, 2005; Xie et al., 2010; Vanneste et al., 2011). The initiation of stomata patterning programs rely on the activation of small secreted peptides, EPIDERMAL PATTERNING FACTORS 1 and 2 (EPF1, EPF2) and STOMAGEN (STOM) (Hara et al., 2007, 2009; Hunt and Gray, 2009; Hunt et al., 2010; Sugano et al., 2010). It is also regulated by the function of several leucine-rich repeat receptor kinases (LRR-RLKs) from the ERECTA family, ERECTA (ER) and ERECTA-like 1 and 2 (ERL1 and 2) as well as by LRR-receptor-like protein TOO MANY MOUTHS (TMM) (Nadeau and Sack, 2002; Shpak, 2005; Hunt and Gray, 2009). EPF1 and 2 inhibit stomata differentiation notably by a negative feedback regulation of the bHLHs expression (Hara et al., 2009; Hunt and Gray, 2009; Lee et al., 2012; Horst et al., 2015). STOM in contrary promotes stomata development by interfering with EPFs function (Ohki et al., 2011; Lee et al., 2015). TMM and the RLKs from the ER family negatively regulate stomatal differentiation by activating a mitogen-activated protein kinase (MAPK) signaling cascade including MAPK and YODA, which ultimately lead to the inhibition of SPCH activity (Nadeau and Sack, 2002; Shpak, 2005; Lampard et al., 2008, 2009; Bergmann, 2014). The spatial distribution of these factors, which are regulated by various feedback mechanisms, will control the spacing and density of stomata within the leaf (Zoulias et al., 2018).

### Genetic Control of Leaf Shape

**FIGURE 2:**
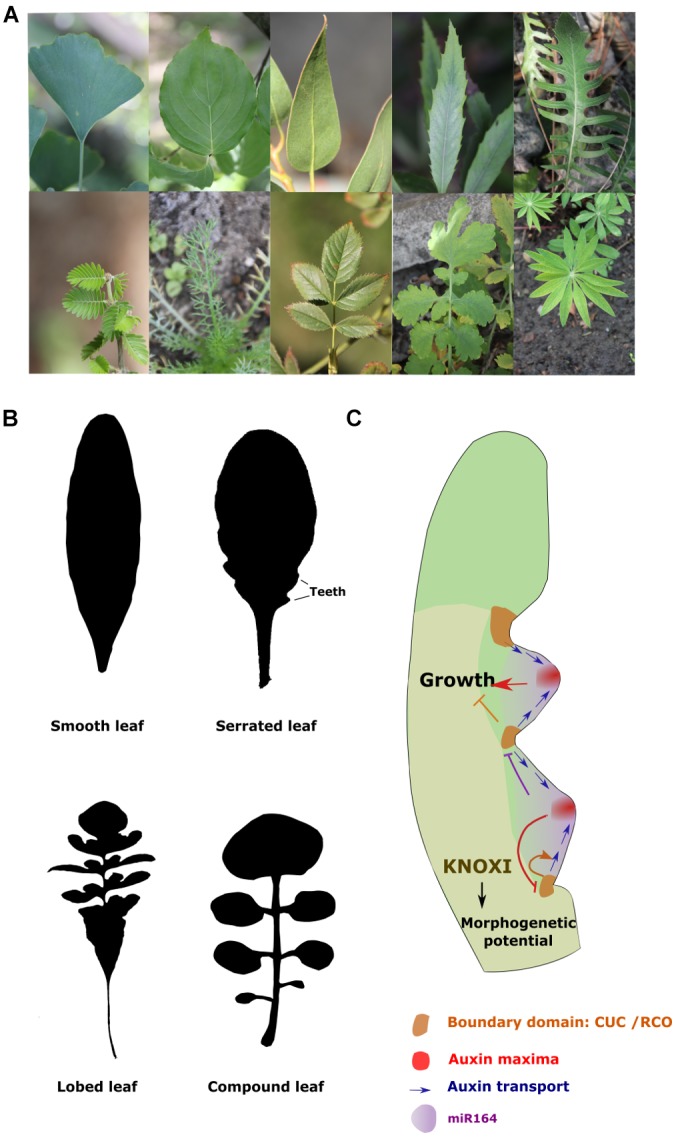
Genetic control of leaf margin dissection. **(A)** Photographs illustrating the diversity of leaf shapes in flowering plants. On the top row from the left to right are examples of simple leaves from: *Cornus kousa* (Cornaceae), *Ginkgo biloba* (Ginkgoaceae*), Lomatia arborescens* (Protoecea), *Serratula radiata* (Asteraceae). On the second row from the left to right are examples of highly dissected leaves from: *Porlieria hygrometra* (Zygophyllaceae) and *Chamomilla recutita* (Asteraceae), *Rosa nutkana* (Rosaceae), *Chelidonium majus L.* (Papaveraceae) and *Lupinus polyphyllus* (Leguminosae) are shown. **(B)** Examples of the main types of leaf margin dissection in plants. The silhouette of a smooth leaf (*Neslia paniculata*), a serrated leaf (*Arabidopsis thaliana*), a lobed leaf (*Capsella rubella*) and a compound leaf (*Cardamine hirsuta*). **(C)** Model of the genetic control of leaf margin dissection: the activation of *CUC* genes will lead to a repolarisation of PIN1 and, thus, to convergent flows of auxin. The resulting high auxin signaling together with miR164 will inhibit *CUC* and promote growth. At the sinuses, CUC and RCO inhibit growth. The formation of compound leaves relies on the maintenance of morphogenetic potential in the leaf primordia by KNOXI.

Leaf shape varies tremendously among flowering plants (Figure 2A) (Bharathan et al., 2002; Bar and Ori, 2014). They can be composed of a single unit with a continuous margin as in the plant model *A. thaliana*, in which case they are said to be ‘simple’ (Figure 2B). But they can also be more complex, such as the compound leaves of tomato, in which the margins are divided into small units called leaflets (Figure 2B). Many intermediate forms varying in the degree of leaf margin dissection can also be found in nature. The geometry of the leaf contours is also highly variable in flowering plants. This has been proposed to result from variation in the spatiotemporal activities of the different leaf meristems (plate, margin and thickening meristem) as well as in the orientation of cell divisions and elongation during leaf development (reviewed in Tsukaya, 2018). The development of compound leaves was proposed to depend on the morphogenetic competence of the leaf margin (Hagemann and Gleissberg, 1996; Maugarny-Calès and Laufs, 2018). In agreement with that, the ability of many species to develop compound leaves is associated with the recruitment of KNOXI expression within the leaf primordia (Hay and Tsiantis, 2006; Shani et al., 2009). KNOXIs appear to maintain meristematic activity through the activation of CK signaling (Shani et al., 2010; Bar et al., 2016). The pattern of leaflet formation as well as of the dissection of the leaf margin is dependent on the function of the NAM/CUC-Auxin module (Barkoulas et al., 2008; Blein et al., 2008; Efroni et al., 2010; Kawamura et al., 2010; Koenig and Sinha, 2010; Bar and Ori, 2014). In *A. thaliana*, it has been proposed that the dissection pattern in the leaf blade is established through a negative feedback loop between auxin and the boundary gene *CUP-SHAPED COTYLEDON 2* (*CUC2*) (Figure 2C) (Bilsborough et al., 2011). According to this model, *CUC2* triggers the intracellular localization of the auxin efflux carrier PIN1 away from its expression domain, creating an auxin convergent flux leading to the formation of auxin maxima at the leaf margin. High auxin response together with the expression of the micro RNA miR164, in turn, inhibit the expression of *CUC2* (Nikovics et al., 2006). These dynamic relationships create a succession of auxin maxima and minima along the leaf margin that mark locations of blade growth promotion and inhibition respectively. This system is also reinforced by a receptor-ligand system that contributes to restraint high auxin response at the tip of the developing teeth. High auxin signaling activates the expression of an ERECTA receptor-kinase but represses its ligand EPIDERMAL PATTERNING FACTOR-LIKE 2 (EPFL2) whose expression is present at the teeth base where auxin signaling is weaker (Tameshige et al., 2016). In these peripheral cells, the ligand will be able to interact and activate its receptor kinase initiating a signalization cascades leading to the repression of auxin response. Genes involved in auxin signaling will, therefore, be essential to mediate the differential growth patterns along the leaf blade (Koenig et al., 2009; Wang et al., 2011; Abley et al., 2016; Ben-Gera et al., 2016). *Indoleacetic acid* genes (*IAA 8* and *9*) have been shown to inhibit auxin response at the sinuses of the teeth promoting their separation and the dissection of the leaf blade margin (Koenig et al., 2009). Similarly, auxin transporters PIN1, AUX1 and LAX1 are essential to the patterning of leaf growth (Bilsborough et al., 2011; Kasprzewska et al., 2015; Abley et al., 2016). This mechanism appears to be conserved among species and to also contribute to the elaboration of more complex leaf forms. *CUC3* and *PIN1* are for instance required from the separation of leaflets in many species with compound leaves (Barkoulas et al., 2008; Blein et al., 2008; Koenig et al., 2009). In contrast GA inhibits leaflet formation most likely by limiting KNOXI function and promoting the transition toward cell expansion. Repressing GA signaling at early stages of leaf growth is, therefore, essential for the elaboration of complex leaf shapes (Jasinski et al., 2008).

Three HD-ZIP homeobox transcription factors encoded by the *REDUCED COMPLEXITY (RCO)* locus have been shown to also play a central role in growth patterning. This locus has evolved through two successive gene duplication events which have both been followed by a functional divergence during which each copy has acquired the ability to regulate growth in different areas of the leaves (Vlad et al., 2014; Streubel et al., 2018). Their function is essential to inhibit growth at the sinuses of leaf teeth, lobes or leaflet primordia (Sicard et al., 2014; Vlad et al., 2014; Tameshige et al., 2016).

## How Leaves Adapt to Changes in Environmental Variables?

Despite the fact that leaf development is tightly controlled at the genetic level, the final shape and dimensions of this organ will also be adjusted based on ambient conditions (Tsukaya, 2005). Not only severe stress conditions will influence leaf development and morphology but also discreet changes in environmental factors. In the following section, we discuss the influence of the major environmental parameters on leaf morphology including growth patterning and anatomical features. Although many of these variables are not totally independent in nature, we focused on what is known about their individual effect on leaf development. We limited our discussion to environment signals that are known to induce a developmental response likely to be adaptive and for which molecular information regarding their integration into GRNs is available. We then describe one of the most striking examples of environmentally induced plasticity in plants - the case of heterophylly in aquatic plants.

### Light: Quality and Intensity

Photosynthesis efficiency will depend on the amount of light captured by the plant. As a result, developmental programs will be adjusted to either maximize light capture or minimize the impact of stress conditions.

**FIGURE 3:**
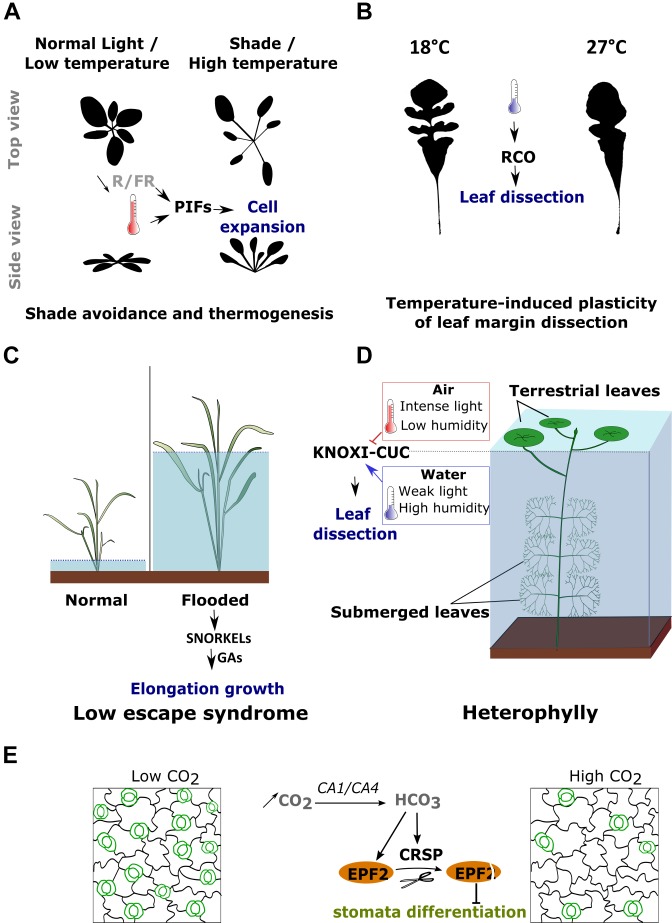
Example of plant developmental responses to changes in environmental conditions. **(A)** Both shaded light (i.e., a reduction in the red to far-red (R/F) photon ratio) and an increase in temperature induce the elongation of the petiole, a reduction of leaf area and an upward movement of the leaves. These responses are mediated by the activation of PIF transcription factors. **(B)** The increase of leaf margin dissection induced by cold in the genus *Capsella* is mediated by the transcriptional activation of *RCO*. **(C)** Deepwater rice species are able to activate elongation growth depending on the water level. In flooded conditions, *SNORKEL 1* and *2* are activated and promote cell elongation through GA signaling. **(D)** Some aquatic species are able to develop two types of leaves each adapted to either submerged or terrestrial conditions. The switch between leaf forms is triggered by several environmental factors including light, temperature and humidity. The decrease in leaf margin dissection in terrestrial conditions is associated with the inhibition of the *KNOXI-CUC* gene modules. **(E)** An increase in CO_2_ induces a decrease in stomata (outlined in green) density through the activation of *EPF2* and of the protease *CRSP*. CRSP cleaves and activates EPF2 which, then, inhibits stomata differentiation.

One of the best described developmental responses to changes in light conditions is the shade avoidance syndrome (SAS) (Figure 3A) (Casal, 2013; Pantazopoulou et al., 2017). Shade is perceived by plants through a reduction in the red (660 nm) to far-red (730 nm) (R/FR) photon ratio. Plants respond to shade by inducing an exaggerated elongation of the stems and petioles, an upward bending of the leaves named hyponasty and, in most cases, a reduction of the leaf blade area. The upward movement of the leaves is achieved by differential cell elongation rates between the lower and upper side of the leaves, while the lamina area is reduced by modulating cell proliferation during leaf development (Carabelli et al., 2007; Casal, 2013). The intensity of this response is quantitative and inversely proportional to the changes in R/FR ratio (Morgan et al., 1980; Cole et al., 2011). This response allows elevating the position of the foliage in order to maximize light capture (Casal, 2013).

In contrary, when the levels of harmful wavelengths increase, plants will tend to avoid extensive exposure to solar radiation. An increase in UV-B, for instance, will lead to the downward curling of the leaves (also known as epinasty), a reduction of the leaf area and an increase in the density of trichomes (Dotto and Casati, 2017). The effect of UV-B on leaf size is species-specific but generally caused by both a reduction in cell proliferation and an increase in cell expansion. The latter is associated with an increase number of endoreduplication cycles during cell expansion (Hase et al., 2006). These observations together with the fact that the geographic distribution of UV-B could efficiently predict the ploidy levels in *A. thaliana* led to the proposal that the increase in endopolyploidy may serve as a protective mechanism against the cytotoxic effect of UV-B radiation (Hase et al., 2006).

Other aspects of leaf anatomy are also affected by light quality and/or intensity. Stomata density was shown to decrease in the dark and increase in high light (Lake et al., 2001; Casson et al., 2009). High light intensities also lead to an increase in leaf thickness mainly due to both an increase in the number of palisade-cell layers (as result of an altered ratio between periclinal and anticlinal divisions) and to the elongation of palisade cells along the thickness axes (Yano and Terashima, 2004; Tsukaya, 2005). The shape of the leaves is also affected by light with an increase of leaf margin dissection in ‘sun’ versus ‘shade’ most likely as an adaptation or consequence of hydraulic limitation (Nicotra et al., 2011).

### Temperature and Leaf Growth

Ambient temperatures will fluctuate considerably during the plant life cycle or over generations. Yet, because leaves have an important function in thermoregulation through transpiration and the regulation of the boundary layer (see below), it may be essential for the plants to adjust their leaf morphology to ambient temperature (Nicotra et al., 2011).

A generic response of plants to changes in temperature occur when they are exposed to high temperatures (Erwin et al., 1989; Quint et al., 2016). This induces a suite of changes in plant phenotypes known as photomorphogenesis, which present many reminiscent features of the developmental changes induced by a low R/FR ratio (Figure 3A) (Casal and Qüesta, 2018). Plants will adapt their morphology to high temperatures by inducing the elongation of the hypocotyl, stem and petioles, as well as a hyponastic growth. This is also associated with a decrease of the leaf thickness and an increase in the density of stomata. Overall, this response is believed to improve the evaporative cooling capacity of the plant, by promoting heat dissipation and by limiting the direct sun exposure through the upward bending of the leaves (van Zanten et al., 2010; Crawford et al., 2012; Bridge et al., 2013; Ibañez et al., 2015).

The level of leaf margin dissection has for long been shown to correlate with ambient temperature. Plants growing in cold and drier climates tend to develop leaves with irregular margins characterized by pronounced sinuses, while plants growing in more tropical, warm and humid conditions display smoother outlines (Bailey and Sinnott, 1916; Webb, 1968; Wolfe, 1978, 1993; Royer et al., 2009; Peppe et al., 2011; Chitwood et al., 2015). This correlation suggested a direct relationship between temperature and leaf shape and is such that, the level of the leaf dissection has been used as an indicator for predicting paleoclimate (Givnish, 1979; Wolfe, 1993, 1995; Wilf, 1997; Wilf et al., 1998; Little et al., 2010). While most these interpretations are purely correlative, they are now supported by several studies that have directly tested the effect of temperature on leaf shape (Figure 3B) (Nakayama et al., 2014; Sicard et al., 2014). Why such correlation exists and what is the adaptive value associated with the plasticity of leaf margin dissection is still debated (Nicotra et al., 2011; Chitwood and Sinha, 2016). Leaf dissection has been shown to positively influence the expression of genes involved in photosynthesis, the leaf photosynthetic activity, as well as the overall seed production (Chitwood et al., 2013; Vuolo et al., 2016; Andres et al., 2017). Consistently with these observations, leaf dissection has been proposed to improve photosynthesis and transpiration during the growing season when climatic conditions are not optimal (Baker-Brosh and Peet, 1997; Royer and Wilf, 2006). Yet, it is unclear how leaves influence these parameters especially in a temperature dependent context. The effect of margin dissection on leaf performance may be associated with the fact that it reduces the area of the lamina compared to the quantity of conducting veins, therefore reducing leaf hydric resistance and improving its conductance (Brodribb et al., 2010; Nicotra et al., 2011). It has also been proposed that leaf dissection may reduce the so-called leaf boundary layer, a thin layer of air at the surface of the leaf where the air flow is considerably reduced and thus where heat transfers only occurs through molecular diffusion, thereby improving leaf thermoregulation at lower temperatures (Schuepp, 1993; Nicotra et al., 2011).

### Water Availability

Plant fitness depends on their ability to optimize water usage efficiency (i.e., carbon gain per units of water loss) which is influenced by, among other things, leaf anatomy and morphology, stomatal conductance, transpiration and the allocation of growth resources to shoot or root (Nicotra and Davidson, 2010). To avoid desiccation or in contrary to limit the oxidative stress caused by water excess, plants have to adapt their development to water availability in the surrounding environment.

Plants response to dry conditions is very complex and will depend on the severity of the stress and on the developmental stages of the plant. In all cases, plants will try to optimize water uptake and limit losses (Mizutani and Kanaoka, 2017). The parameters that influence water retention include the composition, structure and shape of the cuticle, the density and opening of stomata as well as the size of the boundary layer at the surface of the leaf (Mizutani and Kanaoka, 2017). Under moderate drought stress, plants respond by reducing shoot growth most likely to save water and energy resources that they invest in root growth to maximize water intake as well as in reproduction (Claeys and Inze, 2013). The thickness of the cuticle and wax layer increase in dry conditions while the surface area of the leaf decreases (Nawrath, 2006; Goodwin and Jenks, 2007; Wang et al., 2014). At the cellular level, this reduction in leaf expansion is due to a repression of both cell proliferation and elongation (Baerenfaller et al., 2012; Dubois et al., 2017).

Stomata density is positively correlated with humidity (Bakker, 1991; Fanourakis et al., 2016). While a humid environment allows plants to maximize growth by exploiting photosynthesis, an excess of water, such as flooding, will have a negative impact on plant development. In highly humid conditions, the reduction of gasses (O_2_ and CO_2_) diffusion and the oxygen shortage will impair photosynthesis and respiration leading to a strong decrease in leaf growth and to oxidative stress (Jackson, 2002; Bailey-Serres and Voesenek, 2008; Sasidharan et al., 2017). Some plant species, such as deepwater rice, have nevertheless evolved the ability to tolerate flooding (Bailey-Serres and Voesenek, 2008). This tolerance is achieved through the use of an avoidance strategy called the low escape syndrome (LOES) (Figure 3C). This response includes the elongation of petioles and stems, the development of thinner leaves with thinner cell walls as well as the movement of the chloroplasts toward the leaf surface. The accelerated growth most likely allows the leaves to quickly reach a less humid environment while the anatomical changes are believed to diminish the resistance for inward gas diffusion and thus improve underwater photosynthesis (Bailey-Serres and Voesenek, 2008).

### A Case of Study: Heterophylly in Aquatic Plants

One of the most striking examples of environmentally induced plasticity of leaf morphology is observed in several aquatic plants. During their life cycle, these plants will first grow under water before reaching the surface where they continue to develop new organs but in a thereafter terrestrial environment. At this point, the plants will be challenged by a completely new type of environment that differs in an all sets of parameters (Yano and Terashima, 2004; Wanke, 2011). Many aquatic plants have adapted to this transition by inducing drastic developmental changes that will allow matching their leaf morphology and anatomy to totally different requirements, ensuring an optimal metabolism and fitness (Figure 3D) (Nakayama et al., 2012). Underwater, these plants develop highly dissected leaves that are also characterized by an increased hydrophobicity, a reduction of leaf thickness and stomata density (Iida et al., 2016; Li et al., 2017). When the SAM reaches the surface of the water, the change in environmental conditions will trigger a ‘reprogramming’ of leaf development, increasing their thickness and stomata density while promoting lamina growth and the production of smoother margins. Several environmental signals were shown to contribute to triggering these changes. An increase in light intensity, a raise in temperature or a reduction in humidity, are able to individually induce the development of terrestrial leaves. These developmental decisions appear to be taken very early during leaf organogenesis (Goliber and Feldman, 1990; Bruni et al., 1996; Kuwabara and Nagata, 2006). In North American lake cress, the high dissection of the aquatic leaf form is caused by the maintenance of cell proliferation in the basal part of existing leaflets leading to the emergence of new leaflets at the expense of the leaf margin expansion. The transition toward the terrestrial leaf form is associated with a change in the spatial distribution of cell proliferation which becomes more uniformly distributed throughout the leaf primordia (Nakayama et al., 2014).

## Molecular Integration of Environmental Signals

The above examples indicate that plants are able to sense environmental signals and to modify their developmental program accordingly. This raises the questions of what are the sensory mechanisms allowing plants to perceive their environment, what are the molecular mechanisms in place to integrate environmental information into ontogenesis, and how are multiple signals translated into a clear developmental decision? In this part, we review recent studies that have improved our understanding of these questions.

### “Long-Range Signals” Coordinate Developmental Changes Upon Environmental Fluctuations

One of the general features that stand up from these studies is that environmentally induced changes during leaf development are generally caused by long-range non-cell autonomous signals. Many studies have suggested that leaves do not need to be directly exposed to a change in environmental parameters to modify their development. These changes can be sensed locally by mature leaves and transmit to the SAM where new leaves are developing. For instance, exposing mature leaves to high concentrations of CO_2_ or different light conditions was sufficient to reduce the opening and number of stomata in younger leaves (Lake et al., 2001). Similarly, in the aquatic plant *Rorippa aquatica* (Lake cress) subjecting a single mature leaf to a temperature higher than the ambient temperature led to a decrease of the complexity of the margins in the newly developing leaves (Nakayama and Kimura, 2015). During flooding, a long distance signal travels from the root to the shoot where it regulates leaf growth (Jackson, 2002). These results indicate that, in many instances, leaves themselves or other parts of the plants are able to sense changes in the environment and to generate a systemic signal that will modify the developmental programs sculpting the new organs. This may, therefore, suggest an important role for diffusing molecules such as phytohormones in the transmission of these signals.

### Environment-Dependent Transcriptional Regulation of Specialized Regulators or ‘Gene Switches’ as a Trigger of Developmental Responses

An important question toward the understanding of phenotypic plasticity is what are the molecular mechanisms that have evolved to induce specific developmental responses to particular changes in environmental variables. The survey of the literature on this topic indicates that in most cases these responses are mediated by transcriptional changes of key regulatory hubs (Figure 3).

#### Environmental Regulation of Stomata Patterning

CO_2_ concentration regulates stomatal development and an increase in its concentration leads to a reduction of stomata density in *A. thaliana* (Figure 3E). The concentration will be relayed by the activity of two carbonate anhydrase CA1 and CA4, which catalyze the conversion of intracellular CO_2_ into bicarbonate HCO_3_^-^ (Engineer et al., 2016). When CO_2_ concentration increases, the increase of intracellular HCO_3_^-^ will activate the expression of an epidermal patterning factor EPIDERMAL PATTERNING FACTOR 2 (EPF2) and the CO_2_ RESPONSE SECRETED PROTEASE (CRSP) (Engineer et al., 2014). EPF2 is known to interact with an ERECTA receptor kinase and to regulate the differentiation of the protodermal cells into stomata. After being transcribed EPF2 is, however, not yet active and needs to be cleaved by CRSP to be activated (Engineer et al., 2014). The coactivation of both EPF2 and CRSP by CO_2_ allows the inhibition of stomata differentiation, establishing a link between patterning regulators and environmental variables and providing a means to regulate stomatal density based on the concentration of atmospheric gasses. High temperature also leads to a decrease in stomatal density (Lau et al., 2018). In this case, the inhibition of stomata differentiation is, however, mediated by the repression of the bHLH transcription factor *SPCH*, which plays a central role into committing the precursor cells to a stomatal fate (Lau et al., 2018).

Heterophylly in aquatic plants is also associated with drastic changes in stomata density. For instance, *Ranunculus trichophyllus* develops thin cylindrical leaves underwater, which are characterized by a lack of stomata and adaxial-abaxial polarity. Its terrestrial leaves, however, resemble common leaves and have a broad margin as well as fully differentiated stomata (Kim et al., 2018). An aquatic environment induces an overproduction of the plant hormone ethylene leading to the activation of the putative transcription factor *ETHYLENE INSENESITIVE3* (*EIN3*), which in turn ectopically activates the expression of the *KANADI* abaxial genes and inhibits the expression of *STOMAGEN* (*STO*), a central regulator of stomata density and of *VASCULAR-RELATED NAC-DO-MAIN7* (*VND7*), a regulator of vascular development. The deregulation of these patterning genes induces the loss of adaxial-abaxial polarity and lamina differentiation. When the SAM reaches the terrestrial condition, it promotes the production of another plant hormone, the Abscisic Acid (ABA) leading to the activation of *ABSCISIC ACID INSENSITIVE3* (*ABI3*), which in turn activates the adaxial genes, homeodomain-leucine zipper III (*HD-ZIPIIIs*) as well as *STO* and *VDN7*. The expression of these genes finally leads to the establishment of leaf shape polarity as well as the differentiation of vascular system and stomata. Therefore, environmentally induced changes in hormone homeostasis can regulate developmental decisions in plants.

#### Environmental Regulation of Leaf Size

Leaf size is influenced by many environmental signals. Yet, only a few studies have identified the molecular mechanisms which relay environmental signals into the genetic networks controlling growth.

Growth inhibition due to water limitation (e.g., during drought and high salinity stress) is also highly dependent on the transcriptional activation of central regulatory modules. The decrease in leaf area is in part mediated by the activation of two AP2/ERF transcription factors, DEHYDRATION-RESPONSIVE ELEMENT BINDING PROTEIN2A (DREB2) A and B (Sakuma et al., 2002, 2006a,b). Under normal conditions, *DREB2A* is inhibited by the transcription factor GROWTH-REGULATING FACTOR7 (GRF7) (Kim et al., 2012). A decrease in water availability releases this repression leading to *DREB2A* expression and growth retardation. While the underlying mechanism is unclear, this activation is known to arise only within few minutes after a stress signal and is therefore likely to constitute an early response (Sakuma et al., 2006b). Recently the SIAMESE-RELATED (SMR) proteins were also shown to regulate leaf growth in response to water deficit (Dubois et al., 2018). SMRs are involved in the regulation of cell cycle progression through the inhibition of the CYCLIN DEPENDENT KINASE (CDK) proteins (Yi et al., 2014). CDKs, as for instance CDKA, interact with multiples CYCLINs (CYCs) to regulate cell cycle progression. SMRs contribute to inhibiting CYC/CDK complexes by interacting with CDK proteins (Yi et al., 2014). While SMRs contribute to cell cycle progression in normal conditions, they seem to be also involved in the environmental regulation of leaf development. For instance, SMR5 and SMR7 are up-regulated by oxidative stress, while SMR1 is both transcriptionally active and post-transcriptionally stabilized under mild drought stress (Yi et al., 2014; Dubois et al., 2018). Activated SMRs can then interact with CDKs reducing leaf growth in response to water deficit (Dubois et al., 2018).

As described above, to survive flooding some plant species have acquired the ability to accelerate growth through the LOES (Figure 3C). In this case, the excess of water caused by flooding leads to a strong increase in the concentration of the phytohormone ethylene, which in turn leads to the activation of the ethylene response factors *SNORKEL1* and *SNORKEL2*. These genes stimulate cell elongation in the stem and leaves by modulating the biosynthesis of gibberellins (Hattori et al., 2009).

High light intensities, and in particular exposure to UV, have also a negative effect on leaf growth. UV-B inhibits cell proliferation by increasing the level of miR396 the micro RNA repressing the GRFs. As we discussed above, the GRFs contribute to maintain cell division during leaf development. The resulting decrease in GRF levels will therefore, limit cell division and reduce the total number of leaf cells (Casadevall et al., 2013; Fina et al., 2017).

These examples show that indeed a large number of environmental factors can influence leaf growth through the transcriptional regulation of genes at the core of molecular processes determining the final leaf dimensions. Our knowledge of how different climatic parameters integrate gene regulatory networks controlling growth is, nevertheless, rather limited and efforts in this direction are grandly needed.

#### The Control of Leaf Geometry by Environmental Factors

Not only the size of the leaves but also their overall geometry can be affected by environmental factors. Indeed, one of the most striking examples of leaf shape plasticity is the heterophylly in aquatic plants. As for many other leaf traits, a change in hormone homeostasis appears to have a central role in activating the switch between leaf morphs (Nakayama et al., 2017). For instance, in *Hygrophila difformis* and *Ranunculus trichophyllus*, the decrease in leaf dissection induced by terrestrial conditions is mediated by an increase in ABA signaling, while Ethylene induces the aquatic phenotype (Li et al., 2017; Kim et al., 2018). However, the nature of the plant hormones activating this switch can differ between species. In the North American Lake Cress, the transition toward terrestrial leaves is associated with an increase of GA biosynthesis. In this case, the terrestrial leaf phenotype can be induced by exogenous GA treatment, while inhibiting GA synthesis leads to a ‘reversion’ toward submerged phenotypes (Nakayama et al., 2014).

In the above-mentioned studies, however, the decrease of leaf margin dissection is consistently associated with a reduction of the expression of the ortholog of the *KNOXI* transcription factors (*SHOOT MERISTEMLESS* (*STM*) and *BREVIPRDICELLUS* (*BP*)) as well as of the boundary gene *CUP-SHAPED COTYLEDON 3* (*CUC3*) (Figure 3D). While different signaling pathways appear to relay the environmental information, the same gene modules seem to mediate the changes in morphology. Additionally, the switch between aquatic to terrestrial leaf form, in both *Hygrophila difformis* and North American Lake Cress, can be induced by an increase in temperature highlighting a link between temperature and growth patterning. Interestingly, another homeobox gene has been shown to regulate leaf complexity in response to environmental signals in non-aquatic species. In the terrestrial *Capsella* genus, the level of leaf margin dissection is also increased by cold temperature (Figure 3B). Here, the change in leaf morphology appears to be mediated through changes in the expression of a class I homeodomain leucine zipper (HD-ZIPI) belonging to the *RCO* locus (Sicard et al., 2014). As *CUC3* and *KNOXIs*, the *RCO* locus plays a central role in regulating leaf complexity in different species and is known to underlie evolutionary changes in leaf morphology (Hay and Tsiantis, 2006; Blein et al., 2008; Shani et al., 2009; Sicard et al., 2014; Vlad et al., 2014; Vuolo et al., 2016). These results, together with the biogeographical correlation between temperature and leaf margin dissection, suggest a close relationship between climate and leaf shape, highlighting homeobox genes as mediators of this interaction (Bailey and Sinnott, 1916; Webb, 1968; Wolfe, 1978, 1993; Royer et al., 2009; Peppe et al., 2011; Chitwood et al., 2015; Chitwood and Sinha, 2016).

#### Heteroblasty and the Metabolic Regulation of Leaf Development

Although environmental signals have been shown to directly impact specific aspects of leaf morphology, some of their effects may be indirect and linked to their influence on resource availability.

An example of such regulation is the control of heteroblasty. Indeed, leaf morphology changes considerably during the plant life cycle. In *A. thaliana* as the plant matures, the leaves become more serrated, longer and trichromes start to develop on their abaxial side (Poethig, 2013). In the early eighteen century, Goebel firstly hypothesized that the transition from juvenile to adult leaf forms was triggered by a change in the nutritional status of the shoot apex (Chamberlain, 1908). Several studies have since supported this hypothesis by demonstrating that treating juvenile plants with sugars promotes the development of larger and more dissected leaves (Chamberlain, 1908; Allsop, 1954; Rao, 1966). Indeed sugars, such as glucose, act as signaling molecules to repress, both transcriptionally and post-transcriptionally, the micro RNA miR156s (Yu et al., 2013). miR156s are known to induce the degradation of several SQUAMOSA PROMOTER BINDING PROTEIN-LIKE (SBP/SPL) transcription factor mRNAs, which promote the expression of adult traits (Yang et al., 2013). The reduction of miR156 by sugar leads to an increase in SPL proteins, promoting their interaction with TEOSINE BRANCHED 1/CYCLOIDEA/PCFs (TCPs), which normally interact with and inhibit CUC proteins. In turn, the binding of TCPs with SPLs releases CUCs repression, which are then be able to dimerize and ultimately promote leaf serration (Rubio-Somoza et al., 2014).

Any environmental conditions influencing resource availability and thus the nutritional status of the plants are, therefore, likely to modify the timing of heteroblasty through this pathway.

### Sensory Mechanisms and the Induction of Molecular Signals

The discussion in the previous paragraph describes how the environmental signals are integrated into the gene regulatory networks controlling different aspects of leaf growth. But how plants are able to sense changes in environmental variables is on itself a very interesting and important question. While the knowledge on this topic is somewhat limited, some progress has been made in the last years in understanding the mechanisms of light and temperature perception. It has unraveled the molecular nature of the first sensory systems in plant providing plausible mechanisms for the induction of long-range signals.

#### Light Sensing and Signaling

Several sensory photoreceptors involved in the perception of different light wavelengths have been identified. They include phytochromes, cryptochromes, phototropins and UV RESISTANCE LOCUS (UVR8) (Casal, 2013).

**FIGURE 4:**
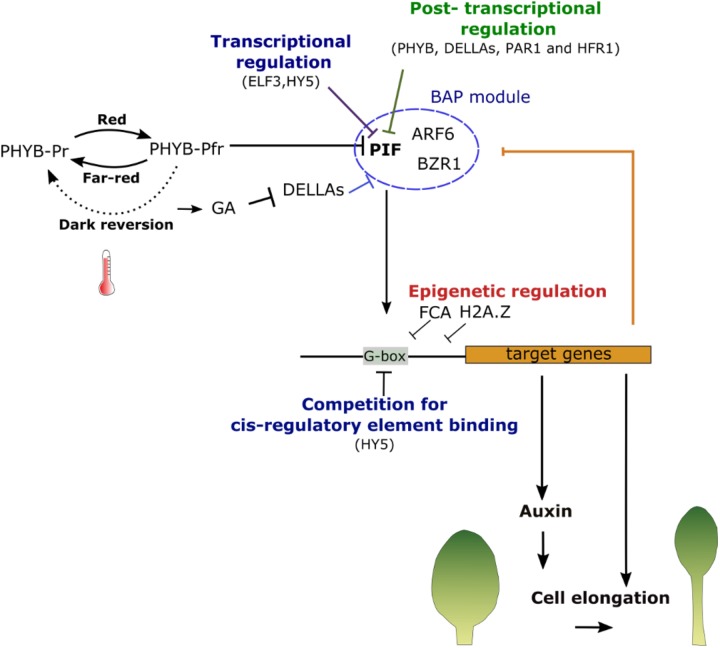
Summary of shade and warm temperatures sensing mechanisms. The photoconversion of phytochromes is dependent on temperature. Both high temperature and a low red to far-red photon ratio will promote the conversion from the active (PHYB-Pfr) to the inactive (PHYB-Pr) PHYB from. This releases PHYB-mediated repression of PIFs, which can then activate their targets. PIFs activities are, nevertheless, regulated spatially and temporally at diverse levels: transcriptionally by transcription factors such as ELF3 and HY5 and post-transcriptionally by interactors preventing its binding to DNA sequences. PIF4 interacts with ARF6 and BZR1 in the BAP module to regulate cell elongation. In normal conditions, all of these factors are bound and inhibited by DELLA proteins. High temperature and shade also increase GA levels leading to the degradation of the DELLAs and the activation of PIFs and their partners. The binding of PIFs to their targets is regulated by the presence of proteins that compete for the same DNA binding sites and by the chromatin state at their promoters. Once activated PIFs will induce the expression of regulatory genes as well as the production of auxin leading ultimately to an appropriate developmental response.

The Phytochrome B (PHYB) plays a central role in the SAS by perceiving changes in the red to far-red photon ratio (R/FR) (Figures 3, 4) (Whitelam and Devlin, 1998). PHYB, like other phytochromes, is a homodimeric photoreceptor that exists in two forms: an inactive form, termed PHYB-Pr, which has a maximum of absorbance in red light; and an active form, named PHYB-Pfr, having a maximum of absorbance in far-red light. The photoconversion from one form to another depends on the R/FR ratio. A low R/FR ratio promotes the conversion toward the inactive form Pf whereas an excess of red light induces the conversion toward Pfr. Once activated the PHYB-Pfr is translocated into the nucleus where it interacts with a family of bHLH transcription factors, the PHYTOCHROME INTERACTING FACTORS (PIF). This interaction induces PIFs phosphorylation and its degradation by the proteasome (Leivar et al., 2012). Shade causes a decrease in R/FR promoting the conversion toward the inactive PHYB-Pr form. PHYB inactivation stabilizes PIFs (in particular PIF4, PIF5 and 7) which will then mediate the SAS (Lorrain et al., 2008; Li et al., 2012; de Wit et al., 2016).

PIFs are believed to regulate growth in response to changes in light through the regulation of hormonal pathways (Müller-Moulé et al., 2016; Procko et al., 2016; Pantazopoulou et al., 2017; Yang and Li, 2017). PIF7, 4 and 5 up-regulate auxin signaling and biosynthesis genes, such as *TAA1* and genes of the *YUCA* family (*YUC 8* and *9*, in particular), directly by binding at their promoters (Hornitschek et al., 2012; Li et al., 2012; Procko et al., 2016). The activation of auxin signaling pathway leads to an increase of cell elongation within the petiole and a decrease of leaf blade growth. The latter is caused by the auxin-dependent activation of the *CYTOKININ OXIDASE/DEHYDROGENASE 6* (*CKX6*) in the developing primordial, which is involved in the degradation of the cytokinins, a promoter of cell division (Carabelli et al., 2007). The PIF4-induced burst of auxin production is, however, transitory. During long exposures to shaded light, auxin levels decrease while the sensitivity of the growth response to auxin concentration increase (Pucciariello et al., 2018). This altered auxin sensitivity is likely to be mediated by changes in the basal levels of key regulators of auxin growth response including the PIFs themselves. At long-term, environmental signals are therefore able to modify the connectivity within the gene regulatory networks controlling hormonal signaling to maintain a growth response without a constitutive production of the signaling molecule. PIFs also promote cell elongation independently from auxins by binding directly the promoters of genes involved in cell expansion such as *EXPANSIN* and cell-wall remodeling enzymes (De Lucas et al., 2008; Leivar et al., 2012). PIF4 also regulates stomatal density in response to environmental signals by repressing *SPCH* in stomata precursors (Lau et al., 2018).

The function of PIFs in promoting growth in response to environmental signals is complex and regulated at several levels (Figure 4). On one hand, PIFs can enhance SAS by inducing the degradation of PHY-B (Leivar et al., 2008). However, they can also activate a negative feedback loop by promoting the expression of HLH proteins lacking the DNA binding (b) domain, such as LONG HYPOCOTHYL IN FAR-RED 1 (HFR1), as well as PHYTOCHROME RAPIDLY REGULATED 1 and 2 (PAR1 and 2). These proteins will, in turn, interact with PIFs and inhibit their ability to bind to DNA (Sessa, 2005). PIFs abundance is also regulated through different pathways. DE-ETIOLATED 1 (DET1) and CONSTITUTIVE PHOTOMORPHOGENESIS 1 (COP1) stabilized PIF4 while BLADE ON PETIOLE 2, a patterning factor regulating leaf growth along the proximal-distal axis, modulates growth response by targeting PIFs to the degradation by the proteasome (Gangappa and Kumar, 2017; Zhang et al., 2017). The SAS response is also modulated by the bZIP transcription factor LONG HYPOCOTYL 5 (HY5), which antagonized PIF4 through the competitive binding of its DNA targets (Toledo-Ortiz et al., 2014; Gangappa and Kumar, 2017). PIF function is also regulated by other plants hormones. In normal conditions, the DELLA proteins interact with the DNA binding domain of PIFs preventing them to bind to their targets. In addition to auxin, shade also induces the expression of several GA biosynthetic enzymes leading to an increase in GA, which promotes the degradation of DELLAs. This will increase the pools of free PIFs in the cell nucleus and enhance the growth response (De Lucas et al., 2008; Feng et al., 2008). BR are also important for the SAS response (Luccioni et al., 2002). DELLAs were also shown to negatively regulate BR signaling by binding and inhibiting a central regulator of BR responses, the transcription factor BRASSINOZOLE_RESISTANT 1 (BZR1) (Gallego-Bartolome et al., 2012; de Lucas and Prat, 2014). Interestingly, BZR1 is also known to interact with PIF4 to synergistically regulate the expression of common targets (Oh et al., 2012). It is therefore likely that the increase of GA upon changes in light intensity will also activate BR signaling. Among the genes regulated by both of PIFs and BZR1 is the HLH protein PACLOBU- TRAZOL RESISTANCE (PRE), which interacts with PAR1 preventing it to inhibit PIF4 and thus further reinforcing the shade avoidance response (Hornitschek et al., 2009; Bai et al., 2012). The intensity of the response will, therefore, depend on the integration of complex molecular processes centered around major regulators, the PIFs, whose activity relies on a balance between protein degradation, transcriptional regulation, protein interactions and the presence of DNA binding competitors (Yang and Li, 2017).

#### Temperature Sensing and Signaling

The developmental adaptation to high temperature is very similar to the SAS suggesting that they may activate similar gene regulatory networks (Franklin, 2008; Quint et al., 2016). In fact, the two pathways share several regulatory modules and the same sensory systems are involved in the perception of both signals (Casal and Qüesta, 2018).

As outlined above PHYB exists in an active PHYB-Pfr and inactive form PHYB-Pr. While the levels of the different forms are strongly influenced by the R/FR ratio, the PHYB-Pfr also reverts back to the PHYB-Pr in the dark. The rate of this ‘dark reversion’ is dependent on temperature (Hennig and Schäfer, 2001; Klose et al., 2015; Jung et al., 2016; Legris et al., 2016). At high temperature, the rate of this reversion increases reducing the level of active PHYB-Pfr and thus stabilizing PIFs. Furthermore, PHYB is able to bind DNA in a temperature-dependent manner. Many of its binding sites overlap with those of PIFs suggesting that decreased PHYB-Pr may also enhance growth response by allowing PIFs to access and activate their target genes (Jung et al., 2016).

Phototropins (PHOT) are sensitive to blue light excitation and, as phytochromes, they exist in an active or inactive state. Exposure to blue light activates the photosensory light/oxygen/voltage domain of PHOT, which then interacts covalently with a flavin mononucleotide (FMN) leading to the activation of the serine/threonine kinase domain at the N-terminus of the protein. In darkness, this active form is reverted toward the inactive state and as for phytochrome, the rate of this reaction is accelerated by high temperature (Fujii et al., 2017). The temperature sensing by PHOT has mainly been associated with the relocation of organelles (e.g., chloroplast and peroxisome) and the nucleus within the cell upon cold temperature (Ogasawara et al., 2013; Łabuz et al., 2015; Fujii et al., 2017). However, phototropins are known to influence other aspects of phototropic response, including leaf growth and movement, making it very likely that the effect of temperature on the PHOT active form also influences plant growth (Christie, 2007).

While both of these sensing mechanisms are dependent on light, they are self-sufficient and only depend on their own intramolecular properties and not on other proteins. But these are, however, not the only levels where the two signaling pathways are interconnected. The photoreceptor CRYPTOCHROME interacts with PIF4 in a blue-light dependent manner and inhibits its transcriptional activity limiting thermomorphogenesis (Ma et al., 2016). Similarly, UVs inhibit thermomorphogenesis through the photoreceptor UVR8 dependent inhibition of PIF4 activity (Hayes et al., 2017).

Because of this strong overlap, many of the regulators of light signaling will also influence temperature perception. The regulation of PIFs activity through various mechanisms will also determine the extent of the response to high temperatures (Figure 4) (Quint et al., 2016). Consistently with its function in inhibiting PIFs, HY5 modulates temperature response (Delker et al., 2014). HY5 protein abundance is itself decreased at high temperatures, which contributes to enhance thermomorphogenesis (Toledo-Ortiz et al., 2014). Other proteins that are known to regulate PIF function (e.g., HFR1 or HY5) or abundance (e.g., DET1) during the light response will also influence thermomorphogenesis (Toledo-Ortiz et al., 2014; Hayes et al., 2017). Similarly to light, GA and DELLA proteins have also been implicated in the thermomorphogenesis response (Oh et al., 2014). High temperatures stimulate GA biosynthesis, which induces the degradation of DELLA proteins. PIF4, BRZ1 and the transcriptional regulator AUXIN RESPONSE FACTOR 6 (ARF6) were shown to interact in a complex, termed the BAP module that regulates the expression of genes involved in cell elongation and photomorphogenesis. In normal conditions, DELLAs bind to these proteins and prevent them to interact with their DNA binding site. The high-temperature induced degradation of DELLAs releases this repression and promotes the growth response (Oh et al., 2014). *PIF4* expression is also regulated around the clock with a peak of expression just before dawn. The repression of PIF4 during the night is mediated by the ‘evening complex,’ including the proteins EARLY FLOWERING 3 (ELF3), ELF4 and LUX ARRHYTHMO (LUX0) (Box et al., 2015). ELF3’s affinity for its DNA target decreases at higher temperatures. Increasing temperature will, therefore, release ELF3 repression of PIF4. Polymorphisms at ELF3 underlying natural variation in temperature response suggests that ELF3 constitutes an important regulatory hub with the potential to fine-tune the relationship between environmental signals and developmental responses (Box et al., 2015; Raschke et al., 2015). The downstream targets of PIF4 are, also, shared between the two pathways. In both cases, PIF-dependent up-regulation of auxin biosynthesis genes (e.g., *YUCCA8*, *TAA1*) leads to the activation of auxin signaling and the up-regulation of genes such as EXPASIN and SMALL AUXIN UP RNAs (SAURs) which directly stimulate elongation growth (Franklin et al., 2011; Hornitschek et al., 2012; Li et al., 2012; Procko et al., 2016).

### Chromatin-Based Mechanisms of Environmentally Induced Plastic Response

Because epigenetic mechanisms are able to induce heritable changes in gene expression without altering nucleotide sequences, they have been proposed to play an important role in environmentally induced developmental plasticity (Bossdorf et al., 2008; Aubin-Horth and Renn, 2009; Kelly et al., 2012; Sultan, 2017; Ecker et al., 2018).

As we have discussed above, environmental variables affect the molecular properties of proteins and the rate of biochemical reactions. Temperature, in particular, is likely to influence a large number of processes. For instance, temperature is likely to influence mRNA turnover. If so, it is important to distinguish between a variation in gene expression that arises from a general change in RNA processing (RNA decay, RNA PolII processivity, etc.) and a variation that derives from active regulatory mechanisms (Sidaway-Lee et al., 2014). In *A. thaliana*, increasing temperature has, generally, a positive influence on both transcription and mRNA decay of most genes (Sidaway-Lee et al., 2014). However, genes that diverge from this average response do so mainly due to the modulation of transcriptional rates without major changes in mRNA decay. The difference in transcription between temperature-responsive genes and average-response genes correlates with the presence of specific epigenetic marks at the locus. Common epigenetic marks such as H3K4me3, H3K9Ac or DNA methylation are associated with average-response genes (also called passive response); whereas marks such as H3K27me3 and H2A.Z are associated with non-average responses. Based on these observations Sidaway-Lee and collegues proposed that the composition of the chromatin state may influence, or even mediate, the transcriptional response to temperature (Sidaway-Lee et al., 2014). Several epigenetic states have indeed been shown to play an important role in the regulation of temperature response. Defects in the incorporation of H2A.Z-nucleosomes into chromatin leads to a constitutive thermomorphogenesis suggesting that the presence of H2A.Z-nucleosome contributes to the temperature-dependent regulation of gene expression (Kumar and Wigge, 2010; Kumar et al., 2012). Histone H3 acetylation has also been shown to be necessary for thermomorphogenesis, notably, by activating the transcription of key high-temperature responsive genes including *PIF4* and *YUC8*. In response to warm ambient temperatures, the SANT-domain protein POWERDRESS (PWR) recruits HISTONE DEACETYLASE 9 (HDA9) at *PIF4* and *YUCCA8* loci promoting H3K9 acetylation and their transcriptional activation (Tasset et al., 2018). The genes whose expression where influenced by PWR were shown to be enriched in H2A.Z in their gene body suggesting a link between the two processes (Tasset et al., 2018).

It is still, however, unclear how temperature signals influence chromatin dynamics. Recently, a detailed transcriptomics study together with the study of YUC8 activation upon temperature shifts have started to shed a light on plausible mechanisms (Lee et al., 2014; Cortijo et al., 2017). The RNA binding protein FLOWERING TIME CONTROL PROTEIN A (FCA) is recruited at the *YUC8* locus through its interaction with PIF4 and mediate histone demethylation, which in turn promotes PIF4 dissociation diminishing the growth response (Lee et al., 2014). The heat shock transcription factor, HSFA1 induces rapid changes in gene expression under heat shock but also under warm, non-stressful, temperatures. HSFA1 was found to bind Heat Shock Elements (HSE) in the promoters of heat responsive genes at cold temperature, suggesting that they may contribute to maintaining a ‘poised’ transcriptional state. Post-translational modifications upon an increase in temperature was proposed to trigger the activation of HSFA1 and lead to the recruitment of other transcription factors, chromatin remodellers and/or components of the transcription machinery (Cortijo et al., 2017). Genes rapidly responding to high temperature also have an H2A.Z nucleosome downstream of the HSEs and the temperature dependent eviction of this nucleosome is dependent on HSFA1 (Cortijo et al., 2017). These results suggest that the coordinate effect of HSFA1 and H2A.Z may provide a rapid transcriptional switch. H2A.Z was proposed to promote gene responsiveness and to facilitate the elongation of RNA polymerase II (RNA polII) (Coleman-Derr and Zilberman, 2012; Weber et al., 2014). It is, therefore, plausible that the activation of HSFA1 together with the presence of an H2A.Z nucleosome at its vicinity may facilitate RNA polII elongation and thus rapid transcriptional responses (Cortijo et al., 2017).

These results suggest that both cis-regulatory and nucleosome architecture at promoters might have an influence on gene ‘responsiveness’ (Weber et al., 2014; Cortijo et al., 2017). Because H2A.Z is not only involved in the perception of temperature, such system may be a general feature of environmentally induced transcriptional responses (Sura et al., 2017).

## Conclusion and Perspectives

**FIGURE 5:**
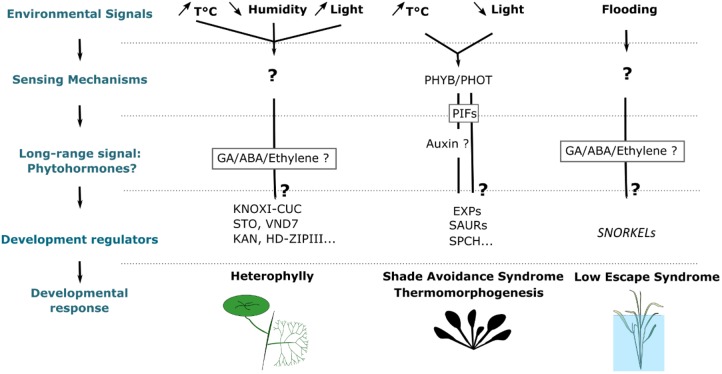
Main phenotypic plastic responses of leaves to changes in climatic variables. This figure summarizes the knowledge and the gaps (labeled with ‘?’) in understanding the molecular mechanism underlying major plastic phenotypic responses in plants. In many cases, they include the integration of several environmental parameters and the activation of long-range signals for which phytohormes are potential candidates. This signal is then translated into appropriate developmental responses through the activation of specific growth regulators. While, much progress has been made to identify these regulators, the perceptions of these signals and the molecular mechanisms conferring the specificity in the developmental response are not very well understood. Based on the current knowledge, the ability of genes to response to environmental triggers may depend on the chromatin state of their promoters.

Leaf shape, size and anatomy are tightly controlled, both temporally and spatially, through complex gene regulatory networks. Many of them include the establishment of negative feedback loops between microRNAs and their targets allowing the formation of spatial domains locally controlling growth pattern along leaf morphogenesis (Yang et al., 2018). While such systems confer sharp and robust boundaries to developmental processes, the multiplicity of the regulatory nodes involved offers many opportunities to fine-tune leaf morphology. Plants have evolved many mechanisms to optimize leaf function according to their surrounding conditions. Based on our literature survey, it appears that environmental signals mostly modify the abundance of key regulators with specialized functions, often acting as developmental switches activating or reinforcing an alternative genetic program (Figure 5). In many instances, this activation is achieved by modifying transcriptional rates, the extent of which is dependent on the architecture of both *cis*-regulatory elements and epigenetic states at the promoters of responsive genes. These transcriptional responses are activated by long-range signals most-likely to induce a coordinated developmental response at the organismal level. Phytohormones are known to be capable of long-distance signaling, and as we have discussed above, they play a key role in mediating environmentally induced developmental responses (Park et al., 2017). They seem, therefore, good candidates to constitute the signaling molecules at the origin of this long-range communications. Changes in hormone homeostasis are induced by sensory systems monitoring different environmental cues. These systems rely on the activation of key regulatory modules capable of inducing a complete transcriptional reprogramming. PIF proteins emerged as a central regulatory hub of environmental sensing in plants, particularly important for thermomorphogenesis and photomorphogenesis. Whether the phytochrome-PIFs module regulates other plastic developmental responses, such as the temperature-induced switch of leaf morph in aquatic species, is still to be determined. Indeed, heterophylly is controlled by multiple environmental and hormonal signals, which are known to be integrated through PIFs activity. Species-specific evolution of connectivity between PIFs-phytochrome and growth patterning genes has the potential to underlie the emergence of new plastic developmental responses. Our knowledge of plant environmental sensing is still limited and in particular, very little is known about how the activation of long-range signals is translated into a particular developmental response. It will be exciting for future research to attempt filling this gap to understand how multiples environmental signals can be integrated into clear and specific morphogenic decisions.

## Author Contributions

MF, SR, and AS wrote the manuscript. MF and AS prepared the figures. All authors read and approved the final manuscript.

## Conflict of Interest Statement

The authors declare that the research was conducted in the absence of any commercial or financial relationships that could be construed as a potential conflict of interest.
